# Role of Mitochondria in Nonalcoholic Fatty Liver Disease

**DOI:** 10.3390/ijms15058713

**Published:** 2014-05-15

**Authors:** Fatiha Nassir, Jamal A. Ibdah

**Affiliations:** Division of Gastroenterology and Hepatology, Department of Internal Medicine, University of Missouri, Columbia, MO 65212, USA; E-Mail: nassirf@health.missouri.edu

**Keywords:** NAFLD, NASH, mitochondria, liver, steatosis, fatty acid oxidation, PGC-1α, sirtuin-1 (SIRT1), sirtuin-3 (SIRT3)

## Abstract

Nonalcoholic fatty liver disease (NAFLD) affects about 30% of the general population in the United States and includes a spectrum of disease that includes simple steatosis, non-alcoholic steatohepatitis (NASH), fibrosis and cirrhosis. Significant insight has been gained into our understanding of the pathogenesis of NALFD; however the key metabolic aberrations underlying lipid accumulation in hepatocytes and the progression of NAFLD remain to be elucidated. Accumulating and emerging evidence indicate that hepatic mitochondria play a critical role in the development and pathogenesis of steatosis and NAFLD. Here, we review studies that document a link between the pathogenesis of NAFLD and hepatic mitochondrial dysfunction with particular focus on new insights into the role of impaired fatty acid oxidation, the transcription factor peroxisome proliferator-activated receptor-γ coactivator-1α (PGC-1α), and sirtuins in development and progression of NAFLD.

## 1. Introduction

**Figure 1.**
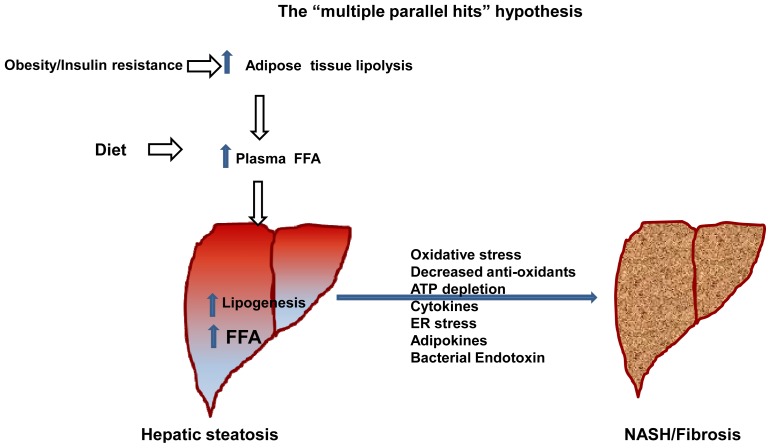
The “multiple parallel-hits” hypothesis of NAFLD: Insulin resistance leads to increased uptake and synthesis of FFAs in the liver, which sensitizes the liver to a series of hits causing liver injury and progression from simple steatosis to NASH [26].

Nonalcoholic fatty liver disease (NAFLD) has evolved as the world’s epidemic and is one of the most common chronic diseases in the United States [1,2]. NAFLD involves a spectrum of hepatic steatosis, nonalcoholic steatohepatitis (NASH), fibrosis, cirrhosis, and hepatocellular carcinoma (HCC) that occurs in the absence of significant alcohol intake and absence of other viral, genetic and autoimmune components. Hepatic steatosis is defined as liver content exceeding 5% of liver weight [3–6]. NASH and alcoholic steatohepatitis (ASH) have similar histopathology but different etiology, and epidemiology [7]. NAFLD affects about 30% of the general population in western society, and is recognized as the most common cause of liver dysfunction worldwide [8]. A 2%–3% subset of patients with NAFLD develop NASH, and 5%–8% of NASH patients develop liver cirrhosis within five years [9,10]. In addition to genetic risk factors, NAFLD prevalence is aggravated by changes in life style including physical inactivity as well as consumption of high fat and high fructose corn syrup diet [11]. NAFLD is considered as the hepatic manifestation of the metabolic syndrome, which is defined by the presence of central obesity, insulin resistance, hyperlipidemia, hyperglycemia, and hypertension [12–14]. NAFLD increases with obesity and type 2 diabetes; it is present in up to 90% of obese individuals [12,15,16]. Insulin resistance is a common causative factor in the pathogenesis of NAFLD and type 2 diabetes, but the underlying mechanisms are far from been understood. NAFLD is a condition that contributes to the metabolic syndrome. Accumulation of the “bad fat” (such as free fatty acids (FFAs), diacylglycerols and ceramides) in hepatocytes in NASH causes insulin resistance, which in turn contributes to type 2 diabetes. Conversely, type 2 diabetes may contribute to NASH, thus creating a vicious circle [17,18]. Type 2 diabetes is associated with liver fibrosis and progression to cirrhosis and HCC [19]. NAFLD is also present in 3%–10% of children; this proportion increases to 50% in obese children in western society [12]. The disease has been described in individuals of all races, in both genders, and also in non-obese subjects [20–22]. There are differences in NAFLD prevalence between various race/ethnic groups. Compared to Caucasian and Hispanic adults, African Americans are protected from the hepatic accumulation of fat [23]. Insulin resistance, oxidative stress, and inflammatory cascades are believed to play a central role in the pathogenesis of NAFLD [24]. Despite the progress gained into understanding how fat accumulates in the liver, the pathogenesis of advanced forms of NAFLD remains to be elucidated. Several hypotheses have been proposed recently. According to the original hypothesis (the “two hit”) hypothesis, NAFLD is a progressive disease in which insulin resistance (“the first hit”) leads to increased FFA flux to the liver. If FFAs are not oxidized or secreted, hepato-steatosis develops [25]. Hepatic steatosis predisposes the liver to “second hits” such as mitochondrial dysfunction, cytokines, adipokines, ER stress and bacterial endotoxins. This original hypothesis has been modified to suggest that NAFLD may be a consequence of parallel “multi-hits” [26]. In this model, insulin resistance leads to increased lipogenesis and increased uptake of FFAs into the liver. Lipotoxicity sensitizes the liver to injury by “multiple parallel hits” (oxidative damage, activation of fibrogenic pathways, activation of hepatic stellate cells, altered expression of adipokines) leading to NASH and fibrosis (Figure 1). More recently a new theory, the “distinct-hit”, has been proposed to suggest distinct pathogenic pathways for hepatic steatosis and NASH [10,27,28]. In general, an association exists between NAFLD and insulin resistance with the exception of some specific genetic conditions affecting lipid metabolism [27,29–32]. A more recent study suggests that metabolic derangements may start early in life, even *in utero*. Exposure to excess fuel in fetal life may lead to NAFLD in the offspring [33].

In this review, we discuss how abnormalities in hepatic lipid metabolism lead to hepatic steatosis. We summarize the current knowledge on mechanisms underlying the development of NASH focusing on the role of hepatic mitochondrial dysfunction. Lastly, we discuss the role of the peroxisome proliferator-activated receptor-γ coactivator-1α (PGC-1α) and sirtuins in the regulation of mitochondrial biogenesis and function in NAFLD.

## 2. Mechanisms Leading to NAFLD

### 2.1. Source of Fatty Acids (FAs) in the Liver

#### 2.1.1. Diet

FAs in the liver originate from the diet, lipolysis of adipose tissue or *de novo* lipogenesis. Dietary fatty acids are absorbed from the small intestine, assembled into lipoprotein rich particles (chylomicrons) and secreted into the blood. High proportions of long chain FAs (LCFAs) in chylomicrons are delivered to adipose tissue, the remaining are taken by the liver [34]. Donnelly *et al.* have demonstrated that about 59% of liver FFAs in NAFLD patients are derived from the circulation, 26% from *de novo* lipogenesis and 15% from the diet [35].

#### 2.1.2. Adipose Tissue Lipolysis

In mammals, excess energy is stored in white adipose tissue (WAT). The major physiological role for WAT is to supply lipid energy to peripheral tissues when needed such as between meals or during physical activity. Lipolysis is the process by which stored triglycerides (TGs) are released as FFAs. This process is regulated by insulin and involves lipases (adipose tissue lipase (ATGL), hormone sensitive lipase (HSL), and monoglyceride lipase), co-lipases, and lipid droplet proteins [36]. Increasing calorie intake such as in obesity causes insulin resistance leading to increased adipose tissue lipolysis, release of FFAs in the circulation and ectopic lipid accumulation.

#### 2.1.3. *De Novo* Lipogenesis

*De novo* lipogenesis is a process by which the cell converts excess carbohydrates into FAs through acetyl-CoA. Hepatic lipogenesis is activated primarily by insulin secreted from the pancreas after a high-carbohydrate meal. Sterol regulatory element binding protein-1c (SREBP-1c) and carbohydrate-responsive element-binding protein (ChREBP) are major transcriptional regulators that induce key lipogenic enzymes responsible for lipogenesis in the liver. SREBP-1c is the master regulator of lipogenic genes and it is regulated by insulin through a phosphoinositide 3-kinase (PI3K)-dependent mechanism that involves the liver X receptor α (LXRα). LXRα promotes the expression of *SREBP-1c* and its target genes fatty acid synthase (*FAS*), acetyl CoA carboxylase (*ACC*), stearoyl-CoA desaturase (*SCD1*) and *lipin* [37,38]. Carbohydrate-responsive element-binding protein is activated by glucose independent of insulin. Although increased flux of FAs to the liver is an important determinant of hepatic steatosis, *de novo* lipogenesis is now considered an important contributing factor to NAFLD development [35].

### 2.2. Fatty Acid Uptake by the Liver

LCFAs can diffuse rapidly across phospholipid bilayers, however accumulating evidence support a facilitated uptake in mammalian cells by a number of transmembrane proteins. FFAs generated by adipose tissue lipolysis under fasting conditions circulate in the plasma bound to albumin. Several transmembrane proteins have been implicated in the transport of plasma FFAs to the liver including plasma membrane FA binding protein (FABPpm), fatty acid transporter protein (FATP), caveolins, fatty acid translocase (FAT)/CD36. Six FA transport proteins (FATP1–6) have been identified in mammalian cells [39]. Two FATPs (FATP2 and FATP5) are expressed in the liver and only FATP5 is liver specific. FATP5 deletion significantly reduces LCFA uptake by hepatocytes isolated from FATP5 knockout animals and lowers hepatic TG [40]. Moreover, adenoviral knockdown of FATP2 or FATP5 reduces hepatic TG accumulation in mice fed a high fat diet [41,42]. Caveolins (caveolin 1–3) are membrane proteins that are found in the membrane structures called caveolae which are important for protein trafficking and the formation of lipid droplets. Caveolin-1 knockout mice exhibit lower TG accumulation in the liver and showed resistance to diet-induced obesity [43]. FAT/CD36, the most studied FA transporter/facilitator, is a transmembrane protein that is highly expressed in many tissues active in lipid metabolism [44]. CD36 plays an important role in facilitating LCFAs uptake and cellular lipid metabolism in rodents and humans. Basal expression of liver *CD36* is low but increases in experimental models of hepatic steatosis, such as genetic obesity and high-fat feeding [2,45]. CD36 upregulation in the liver is associated with insulin resistance, hyperinsulinaemia and increased steatosis in nonalcoholic steatohepatitis [46]. CD36 is also involved in adipose tissue lipolysis; its regulation of adipose tissue lipolysis affects fat accumulation in liver [47]. Adipose tissue *CD36* expression is negatively correlated with intrahepatic TG [48], and CD36 deletion in *ob*/*ob* mice reduces VLDL secretion and aggravates hepatic triglyceride content [2]. The plasma membrane associated FA-binding protein (FABPpm) is identical to the mitochondrial aspartate aminotransferase, an enzyme that functions in maintaining the cytoplasmic/mitochondrial nicotinamide adenine dinucleotide NADH/NAD ratio [49]. FABPs are cytosolic lipid binding proteins that facilitate intracellular transport of FFAs [50]. Among the isoforms identified, LFABP is the highly expressed FABP in the liver. Targeted deletion of LFABP in mice abrogates fasting induced hepatic TG accumulation [51].

Depending on the physiological conditions, these FFAs have multiple destinations in the liver: (1) FFAs are converted into complex lipid species, packaged into very low density lipoproteins (VLDL) and released into the circulation; (2) oxidized by β-oxidation; or (3) esterified into TG and stored as lipid droplets surrounded by lipid droplet proteins within the hepatocyte [52].

### 2.3. Disposal of Hepatic FFAs

#### 2.3.1. Secretion of Hepatic TG

The liver secretes TG in the form of very low density lipoprotein (VLDL). VLDL consists of hydrophobic core lipids containing TGs and cholesterol esters and apolipoprotein B (apoB). The assembly of VLDL in the endoplasmic reticulum requires the crucial interaction between apoB and microsomal triglyceride transfer protein (MTTP) which facilitates the secretion of the lipoprotein particle. Mutations in apoB 100, or in MTTP cause lipid droplet accumulation in the liver [53,54].

Insulin plays an important role in the regulation of VLDL assembly and secretion. Insulin inhibits posttranslational degradation of apoB 100 and inhibits MTP at the transcriptional level [55–58]. Insulin resistance results in increased TG secretion and hypertriglyceridemia; the hepatic steatosis associated with insulin resistance results from increased TG synthesis exceeding liver capacity to secrete TG. Both hypertriglycedemia and hepatic steatosis are observed in NAFLD patients [59]. However, advanced NASH is associated with reduced or complete loss of fat. The mechanisms for fat loss are not understood but may implicate adiponectin [60].

#### 2.3.2. Fatty Acid Oxidation

**Figure 2.**
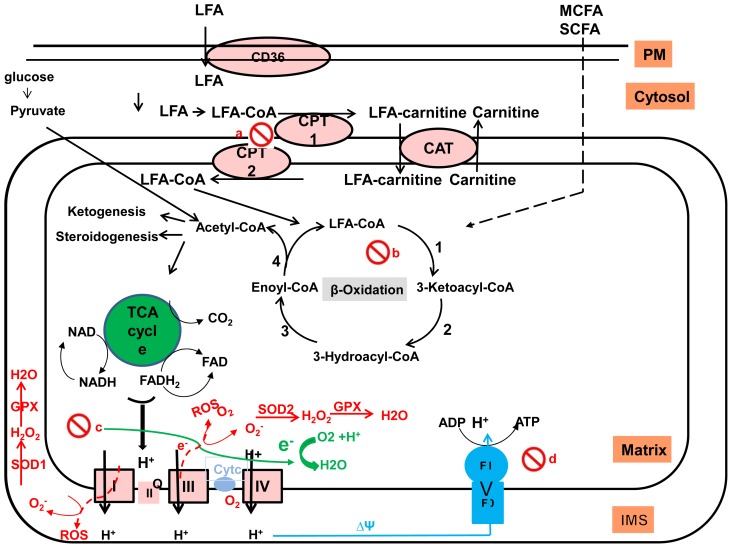
Hepatic β-oxidation: Mitochondrial β-oxidation involves four individual reactions (1–4) to generate NADH or FADH_2_, which are then oxidized to H_2_O by the mitochondrial respiratory chain. The mitochondrial respiratory chain consists of four respiratory complexes (I–IV) involved in the conversion of NADH and FADH_2_ into oxidized cofactors NAD and FAD. Leakage of electrons at complexes I and II results in the formation of superoxide (O_2_^−^) which is then transformed to H_2_O_2_ by superoxide dismutase (SOD1) in the intermediate space and by SOD_2_ in the matrix to H_2_O_2_. Both H_2_O_2_ and O_2_^−^ generated are reactive oxygen species (ROS). Mitochondrial antioxidant enzymes (SOD and glutathione peroxidase GPX) play a role in scavenging mitochondrial ROS. Adapted with permission from [61]. Copyright 1999–2014 John Wiley & Sons, Inc.)

Mitochondria play a central role in energy production (Figure 2). Sugars and fatty acids undergo glycolysis and mitochondrial β-oxidation, respectively to produce acyl-CoA [61]. As demonstrated in Figure 2, the entry of the acyl-CoA into the mitochondria is dependent on the carnitine palmitoyltransferase-1 (CPT-1) present at outer mitochondrial membranes, which catalyzes the formation of acylcarnitine from acyl-CoA and free carnitine. Acyl-carnitines are transported across the inner mitochondrial membrane by CPT-2). Long chain acyl-CoAs are sequentially broken down by β-oxidation cycle into acetyl-CoAs by four reactions that generate NADH and flavin adenine dinucleotide (FADH2): (1) The first reaction involves the membrane associated very-long-chain acyl-CoA dehydrogenases (VLCAD); (2) Hydration of the 2,3 double bond by 2-enoyl-CoA hydratases (membrane associated mitochondrial trifunctional protein (MTP) α-subunit); (3) Dehydrogenation of 3-hydroxy acyl-CoA esters by 3-hydroxy-acyl-CoA dehydrogenases (membrane associated MTP α-subunit, containing LCHAD); and (4) Liberation of acetyl-CoA by 3-keto acyl-CoA thiolases (membrane associated MTP β-subunit). The NADH and FADH2 produced by both β-oxidation and the TCA cycle are used in the final common oxidative phosphorylation system (OXPHOS) to generate ATP coupled with the transfer of electrons along the respiratory chain. FAs are the primary energy substrate for the production of ATP in skeletal muscle and the heart. Under normal conditions, the liver oxidizes FAs mainly when blood glucose is low such as in prolonged fasting and increased physical activity. Hepatic β-oxidation provides ketone bodies (acetoacetate and β-hydroxyacetate), which are important fuel for extra-hepatic organs such as the brain when blood glucose levels are low [62,63].

In the postprandial state, high insulin and glucose favor *de novo* lipogenesis and suppress mitochondrial fatty acid oxidation (FAO) by two mechanisms: Insulin inhibits white adipose tissue lipolysis thereby reducing the flux FFA to the liver. On the other hand, glucose and insulin also regulate the entry of fatty acids into the mitochondria [64]. Insulin facilitates *de novo* lipogenesis through upregulation and activation of sterol regulatory binding protein (SREBP-1c) and induction of acetyl CoA carboxylase (ACC). Glycolysis produces pyruvate which is then transformed within the mitochondria into acety-CoA and citrate. In the cytosol, acetyl-CoA is transformed to malonyl-CoA and then to palmitate by ACC and the fatty acid synthase (FAS), respectively. Elevated malonyl-CoA produced by ACC activity inhibits CPT1 thereby decreasing the rate of β-oxidation by reducing fatty acid entry to the mitochondria. In this scenario, FFAs are directed toward TAG formation and VLDL secretion. Mitochondria are implicated in the oxidative phosphorylation (OXPHOS) and the production of reactive oxygen species, molecules that play an important role in signaling. During the fasting state, glucagon promotes FAO. Glucagon signaling activates AMP-activated protein kinase (AMPK) which in turn inactivates ACC1 and ACC2 by phosphorylation [63]. This blocks the synthesis of malonyl-CoA and is accompanied by activation of malonyl-CoA decarboxylase, which enhances the elimination of malonyl-CoA [63]. These combined effects decrease FA synthesis and activates ketogenesis. PPARα is also essential for glucagon-mediated FAO [65]. PPARα is a FA activated nuclear hormone receptor that plays an important role in the transcriptional regulation of lipid and glucose metabolism [66]. Activated PPARα forms a heterodimer with RXR, which binds to specific DNA sequences known as peroxisome proliferator response element (PPRE). These transcriptional complexes promote the expression of genes that mediate FAO. The fibroblast growth factor 21 (FGF21), a PPARα target, promotes FAO and ketogenesis during fasting [67].

### 2.4. Alterations in Lipid Metabolism Leading to NAFLD

Increasing calorie intake and physical inactivity have caused a worldwide increase in obesity and insulin resistance leading to ectopic lipid accumulation. Insulin resistance and hyperinsulinemia drive *de novo* lipogenesis, and triglyceride synthesis. Increased formation of proinflammatory cytokines and stellate cell activation results in collagen deposition and fibrosis [68]. Dysregulation of lysosomal metabolism increases endoplasmic reticulum stress, leading to cell apoptosis and cell death [69]. Progress has been made in understanding how fat accumulates in the liver, however mechanisms responsible for NASH are still under investigation. The proposed mechanisms for the progression of NAFLD include oxidative stress and induction of proinflammatory cytokines and gut derived bacterial endotoxins [70,71].

## 3. Role of Mitochondria in NAFLD

### 3.1. Mitochondrial Structure

Mitochondria are organelles with a double membrane structure. The outer membrane delimits the intermediate space while the inner membrane delimits the mitochondrial matrix. The structure of the inner membrane is highly complex and consists of the complexes of the electron transport system, the ATP synthase, and transport proteins. The matrix contains a highly concentrated mixture of enzymes involved in the oxidation of pyruvate and fatty acids in the TCA cycle. Human mitochondrial DNA (mtDNA) is found in the matrix and consists of 13 structural genes that encode subunits essential for respiratory complexes I, III, IV, and V of the mitochondrial respiratory chain (MRC) involved in the generation of ATP [72].

### 3.2. Mitochondrial Function

Mitochondria are the power producer of the cell, which plays a central role in the generation of energy from nutrient oxidation. In addition to the role of mitochondria in the oxidation of glucose and fat to produce energy (discussed earlier here), mitochondria play an important role in the generation of reactive oxygen species (ROS) [61] (Figure 2). Hepatocytes are rich in mitochondria; each hepatocyte contains about 800 mitochondria. Fuel oxidation into acetyl-CoA and its subsequent oxidation by the TCA cycle generate NADH and FADH2. The flow of electrons donated from NADH and FADH2 through the ETC is coupled with pumping of protons from the mitochondrial matrix into the inter-membrane space, thus creating an electrochemical gradient across the membrane resulting in ATP synthesis [73] (Figure 2). Excessive flow of electrons to the ETC results in the production of ROS [73]. Mitochondria are a major site for ROS species generation (90%). Under normal conditions, about 1%–2% of mitochondrial oxygen consumption results in ROS production [74]. Excessive production of ROS exceeding the cell’s antioxidant capacity can damage components of the cell such as lipids, proteins and nucleic acids (particularly mtDNA) leading to oxidative stress and ultimately apoptosis. This can be observed in conditions of increased oxidation of FFA such as in NASH [75] and as a consequence of alcohol metabolism in ASH [76].

### 3.3. Mitochondria and NAFLD

Recent evidence suggests that NAFLD might be a mitochondrial disease. Mitochondrial dysfunction contribute to the pathogenesis of NAFLD since it affects hepatic lipid homeostasis, promotes ROS production and lipid peroxidation, cytokine release and cell death [75]. Selected recent published studies linking mitochondrial dysfunction to NAFLD are listed in Table 1. NAFLD has been shown to be associated with mitochondrial paracrystalline inclusions [77,78]. These paracrystalline inclusions have been observed in many mitochondrial myopathies [79]. Uncoupling of the oxidation and the phosphorylation and increased free radical production and lipid peroxidation causes cell injury [79]. ROS production causes lipid peroxidation of mitochondrial membranes which can contribute to impaired mitochondrial function and perpetuate the ROS generation. Oxidative stress also triggers production of inflammatory cytokines, causing inflammation and fibrogenic response. This ultimately results in the development of NASH [80].

Several studies from our group support a role of mitochondrial dysfunction in the development of NAFLD. We have generated mice deficient in mitochondrial trifunctional protein (MTP). Human MTP deficiency causes Reye-like syndrome, cardiomyopathy, or sudden unexpected death [81]. MTP defects were first reported in humans in 1992 [82]. While complete MTP deficiency occurs in about 1:38,000 pregnancies, it is estimated that 1% of the US population is heterozygous for a defect in MTP [83]. Homozygous mice for MTP deletion die shortly after birth [81]. Using heterozygous mice for MTP (HET), we have demonstrated that defects in β-oxidation predispose mice to NAFLD and systemic insulin resistance in an age dependent manner consistent with other studies suggesting a role of mitochondrial dysfunction in the development of NAFLD [84,85]. Aging but not young MTP-HET mice develop hepatic steatosis with elevated alanine aminotransferase (ALT), and increased insulin compared to wild type (WT) control mice. In response to insulin challenge, aging mice display slower rate of glucose disappearance [84]. Hepatic steatosis and insulin resistance develop concomitantly around nine months of age [84]. In addition, aging MTP-HET mice exhibit higher hepatic oxidative stress [84]. The primary defect in mitochondrial fatty acid β-oxidation causes hepatic insulin resistance [84]. We have shown, in subsequent studies that defects in mitochondrial FA β-oxidation (both in isolated mitochondria and in primary hepatocytes) cause hepatic insulin resistance independent of obesity or HFD effect [85]. The hepatic insulin resistance was selective to hepatic glycogen and independent from commonly known factors for insulin resistance such as diacylglycerides and ceramides accumulation in the liver [85].

Using a rat model of obesity and type 2 diabetes, the obese and hyperphagic Otsuka Long-Evans Tokushima Fatty (OLETF) rat [89], we found that reduced hepatic mitochondrial content and function precede development of NAFLD and insulin resistance in these rats, suggesting that mitochondrial biogenesis and function contribute to the development of obesity associated NAFLD [88]. The OLETF rat exhibits insulin resistance at 13–20 weeks of age associated with impaired mitochondrial FA oxidation and hepatic steatosis without significant liver injury. More advanced stages of NAFLD in these rats are observed at 40 weeks of age in association with increased oxidative stress, loss of hepatic anti-oxidative capacity, aggravation of the glycemic control, and ultrastructural changes in mitochondria [88].

**Figure 3.**
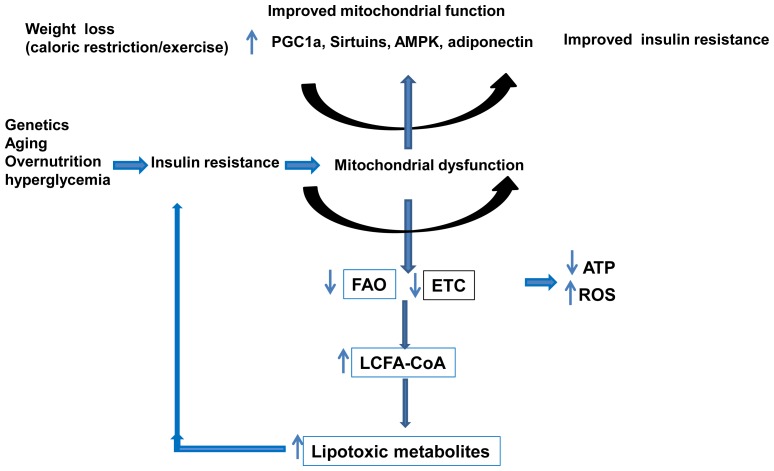
Role of the mitochondria in NAFLD. Impairment of mitochondrial function by genetic factors, aging, and overnutrition causes insulin resistance and mitochondrial dysfunction. Defective mitochondrial β-oxidation causes fatty liver and increases lipid toxic metabolites which may in turn causes insulin resistance, thus creating a vicious cycle between insulin resistance and mitochondrial dysfunction. Improved mitochondrial function by weight loss through caloric restriction and/or exercise improves insulin resistance. Regulators of mitochondrial biogenesis and function include genes such as peroxisome proliferator-activated receptor-γ coactivator-1α (PGC-1α) and sirtuins. AMP-activated protein kinase (AMPK), fatty acid oxidation (FAO), mitochondrial respiratory chain (MRC), long chain fatty acid CoA (LCFA), Adenosine triphosphate (ATP), reactive oxygen species (ROS).

Obesity and particularly visceral obesity are associated with NAFLD. Adiponectin is an adipokine secreted by adipocytes into the circulation that has protective role in obesity related fatty liver disease [60,90,91]. Lower adiponectin levels have been found as an independent risk factor for NAFLD; NASH patients have lower adiponectin levels than controls [92–94]. Adiponectin deficient mice show an increased lipid accumulation due to impaired mitochondrial function [86]. Adiponectin treatment restores the activities of mitochondrial respiratory chain (MRC) through upregulation of uncoupling protein-2 (UCP2) and inhibition of ROS production from mitochondria [86,95]. The role of mitochondrial dysfunction in NAFLD is illustrated in Figure 3.

Studies in both humans and rodents have shown a strong association between ab normal intramyocellular lipid metabolism, mitochondrial dysfunction and insulin resistance. Evidence of mitochondrial dysfunction in skeletal muscle has been shown in type 2 diabetes and insulin resistant subjects [96]. In these subjects, insulin resistance has been shown to be associated with reduced mitochondrial function, decreased mitochondrial size and reduced mitochondrial density, reduced activity of mitochondrial enzymes, lower expression of oxidative phosphorylation genes and lower ATP production.

Similar to the molecular mechanisms observed in hepatic insulin resistance associated with NAFLD, the cellular mechanisms by which intramyocellular lipids cause insulin resistance are not well understood. The relationship between the intramyocellular accumulation of lipids, mitochondrial dysfunction and insulin resistance is complex and a role of mitochondrial dysfunction in skeletal muscle insulin resistance is still to be proven. Several studies have shown a strong relationship between reduced mitochondrial content and activity, intramyocellular lipid content and insulin resistance [97,98]. Reduced mitochondrial content was not associated with changes in the expression of *PGC-1α* (a gene involved in mitochondrial biogenesis) and its target genes [97]. However reduced mitochondrial content in these subjects was associated with lower expression of lipoprotein lipase suggesting a potential role of LPL in the FAs delivery to the muscle and the regulation of mitochondrial content [99]. In addition, moderate weight loss in the resistant offspring was beneficial in improving peripheral insulin resistance without changes in mitochondrial content suggesting that improved insulin resistance was most likely due to reduced FAs delivery to the myocyte during weight loss treatment [100]. The findings, both in the liver and the skeletal muscle, implicate the mitochondria in insulin resistance, type 2 diabetes and NAFLD. Whether changes in mitochondrial energetic efficiency are a causative or adaptive response is still under investigation but a two-way system is plausible (Figure 3).

## 4. Mechanisms Regulating Mitochondrial Function

Proper mitochondrial function is critical for the maintenance of the life of the cell Mitochondria are dynamic organelles; their mass, morphology and positioning in cells are tightly regulated by processes of fission and fusion, biogenesis and mitophagy. Because of their critical role in the cell’s function, mitochondria have been implicated in various metabolic and age related disorders.

### 4.1. Role of the Peroxisome Proliferator-Activated Receptor-γ Coactivator 1 (PGC-1)

A number of transcription factors and co-regulators are involved in the regulation of cellular and mitochondrial metabolism. The transcriptional co-regulators sense changes in metabolism and regulate gene expression accordingly. One of the most characterized co-activators is PGC-1. Three members of the PGC-1 family have been identified: PGC-1α, PGC-1β, and the PGC related co-activator (PRC). PRC is present in all tissues and has been implicated in the regulation of the respiratory chain function [101,102]. PGC-1α and PGC-1β are highly expressed in tissues with high oxidative capacity such as brown fat, heart, kidney, skeletal muscle, and brain where they activate genes involved in cellular energy production [103–105]. Mice with deletion of both PGC-1α and PGC-1β die shortly after birth due in part to cardiac failure [106]. Mice with deletion of either PGC1-1α or PGC-1β are normal under normal conditions but exhibit abnormal response to various physiologic stimuli such as fasting and exercise [107–110]. In addition to their common central role in oxidative metabolism, PGC-1α and PGC-1β regulate specific cellular pathways. PGC-1α is involved in the adaptation to fasting and is involved in gluconeogenesis which is the *de novo* synthesis of glucose in the liver, whereas PGC-1β is involved in the process of *de novo* lipogenesis through activation of the sterol response element binding protein (SREBP1) [37,111,112]. Selective activation of PGC-1β within hepatocytes protects the liver from hepatic steatosis and from progression to fibrosis by inducing mitochondrial oxidative phosphorylation, FAO and TG secretion in the blood stream as well as decreasing oxidative stress caused by dietary-induced steatosis and steatohepatitis [113]. PGC-1α is the most studied and the most characterized PGC1 and has been shown to physically interact with the nuclear receptors PPARα, HNF4 and FXO1 which are essential for the hepatic adaptation to fasting [104]. The liver is a major metabolic tissue responsible for glucose homeostasis in mammals; the brain and red blood cells depend on glucose as an energy source, which is particularly crucial during fasting. Fasting blood glucose levels are maintained through gluconeogenesis, which occurs mainly in the liver. Two hormones control blood glucose levels: insulin and glucagon. Insulin inhibits glucose production in the fed state while glucagon stimulates it in the fasting state. Alteration in the production or the sensitivity to these hormones leads to constant activation of neoglucogenesis and glucose production such as in the case in diabetes. Insulin and glucagon achieve glucose homeostasis through transcriptional regulation of PGC-1α. Glucagon, through cAMP, activates the transcription factor ChREBP which is an essential regulator of PGC-1α. Once activated, PGC-1α co-activates transcription factors required for the PGC-1α-mediated induction of neoglucogenic genes such as HNF4α and FOXO1. Mice with liver-specific deletion of PGC-1α exhibit hepatic steatosis due at least in part to impaired mitochondrial oxidative capacity and mitochondrial dysfunction. We have recently demonstrated that overexpression of hepatic *PGC-1α* and subsequent increases in FAO through elevated mitochondrial content and/or function result in reduced triglyceride storage both *in vivo* and *in vitro* [114]. PGC-1α overexpression in rat primary hepatocytes results in an increase in markers of mitochondrial content and function (citrate synthase, mitochondrial DNA, and electron transport system complex proteins) and an increase in FAO [114]. Exercise also increases hepatic PGC-1α levels in rat models with access to running wheels [115]. Chronic HFD however reduces hepatic PGC-1α levels in sedentary WT mice resulting in reduced hepatic mitochondrial respiration [116].

Both PGC-1α and PGC-1β co-activate a variety of nuclear receptors and transcription factors including PPARs, estrogen-related receptors (ERR), and nuclear respiratory factors (NRF1 and NRF2) to activate expression of genes implicated in FAO and oxidative phosphorylation [117,118]. It is now proposed that PGC-1α is controlling global oxidative metabolism by both regulating mitochondrial biogenesis and the intrinsic properties of the mitochondria. Besides the regulation of PGC-1α expression, PGC-1α activity has also been shown to be regulated by protein modification such as acetylation, methylation, or phosphorylation [119]. PGC-1α is activated by SIRT1 deacetylation and by p38 MAP kinase through phosphorylation [119,120]. The inhibition of PGC-1α is mediated through protein phosphorylation by AKT and Clk2 and through acetylation by GCN5 [119].

### 4.2. Emerging Role of Sirtuins

Sirtuins are a family of NAD-dependent protein deacetylases that are implicated in many cellular and physiological functions including hepatic fatty acid metabolism, mitochondrial function, hepatic gluconeogenesis, insulin secretion and the maturation of fat cells [121–123]. There are seven sirtuins (SIRT1–SIRT7) in mammals that all share the same conserved NAD binding site and catalytic core domain but with different *N* and *C* termini [124]. The different sirtuins have distinct functions and subcellular distribution and expression [124]. SIRT (1, 6, and 7) are localized predominately to the nucleus. SIRT 3, 4 and 5 contain the *N* terminal mitochondrial targeting sequences and reside in the mitochondrial matrix while SIRT2 is mainly cytoplasmic [124]. The most studied sirtuins are SIRT1 (nuclear) and SIRT3 (mitochondrial). These sirtuins have specific molecular targets with SIRT1 playing a role in several processes ranging from cell cycle regulation to energy homeostasis while SIRT3 plays an important role in mitochondrial function [124,125].

#### 4.2.1. SIRT1

In humans and mice, caloric restriction has been shown to reduce glucose and insulin levels and improve insulin sensitivity [126,127]. These beneficial effects of caloric restriction appear to be mediated by SIRT1. SIRT1 regulates central metabolic functions such as cellular fuel metabolism, inflammation, mitochondrial function, and liver regeneration through deacetylation of SIRT1 targets [128]. SIRT1 is upregulated during negative energy balance, as occurs during fasting and caloric restriction and links nutritional status with metabolic homeostasis [129]. Studies have shown that SIRT1 expression in the liver is significantly decreased in an NAFLD model of rats fed a high-fat diet [130,131]. *SIRT1* overexpression in mice with caloric restriction protects mice from developing NAFLD [132]. Indeed SIRT1 transgenic mice exhibit several metabolic benefits that are similar to the caloric restriction phenotype. Mice overexpressing SIRT1 are leaner and resistant to hepatic steatosis and insulin resistance [133]. However, liver specific deletion of SIRT1 promotes hepatic steatosis [134]. It has been shown in another study that liver specific deletion of SIRT1 in mice impairs peroxisome proliferator-activated receptor α (PPARα) signaling and decreases FAO and ketogenesis [135]. This contributes to the high fat induced hepatic steatosis, inflammation, and ER stress in these mice [135]. *SIRT1* deletion in the liver impairs PPARα signaling which is involved in FAO while *SIRT1* overexpression enhances PPARα activity [135]. SIRT1 appears to stimulate PPARα signaling through deacetylation and activation of *PGC-1α* [135,136]. Moreover, hepatic overexpression of the fibroblast growth factor 21 (*FGF21*), a metabolic hormone predominantly produced by the liver that is involved in FAO, in liver specific *SIRT1* knockout mice increases the expression of genes that regulate fatty acid oxidation and decreases fasting-induced steatosis [134].

#### 4.2.2. SIRT3

SIRT3 is the most studied mitochondrial sirtuin, it is a soluble protein located in the mitochondrial matrix. Interestingly, mice lacking SIRT3 appear to be phenotypically normal under basal conditions but show high levels of mitochondrial protein hyperacetylation [137]. Mice lacking SIRT3 are viable and fertile, develop normally, and exhibit no significant differences in body weight, energy balance and adaptive thermogenesis under normal physiological conditions [137]. Recent studies show that 65% of all proteins in mitochondria have at least one lysine acetylated in liver tissue of SIRT3 knockout mice [138]. Several SIRT3 targets have been identified such as long chain acyl-CoA dehydrogenase (LCAD), an enzyme involved in the first reaction of the fatty acid oxidation spiral [139], the mitochondrial enzyme 3-hydroxy-3-methylglutaryl CoA synthase 2 (HMGCS2) which is involved in ketone body formation [140], and the mitochondrial enzyme acetyl-CoA synthase (AceCS) that converts acetate to acetyl-CoA [141]. SIRT3 also regulates the acetylation levels of mitochondrial electron transport Complex I and regulates ATP synthesis [142]. Indeed, ATP levels are reduced by more than 50% in the heart, liver and kidney of mice lacking SIRT3 [142]. Succinate dehydrogenase (one of complex II subunits) has been identified as a direct target of SIRT3 suggesting a role of SIRT3 in the regulation of complex II [143,144].

SIRT3 is upregulated during nutrient distress conditions such as caloric restriction, fasting and nutrient excess. Recent studies in SIRT3 deficient mice have demonstrated that fasting reduces fatty acid oxidation and ATP production, and this was associated with increased hepatic TG content [139]. Fasting induces deacetylation of LCAD and increases its activity [139]. Deacetylation of this enzyme is also induced by *SIRT3* expression both *in vivo* and *in vitro* suggesting that SIRT3 modulates FAO by controlling the acetylation levels of mitochondrial proteins. Interestingly, LCAD deficiency is also associated with accelerated development of insulin resistance and steatohepatitis in mice [145], attributed primarily to lipid accumulation from reduced fatty-acid oxidation [146].

Caloric restriction induces SIRT3 and reduces oxidative stress by SIRT3 mediated activation of SOD2 [138,147]. However, caloric excess such as in obesity and chronic HFD reduces liver SIRT3 activity, impairs mitochondrial function and induces hyperacetylation of various mitochondrial proteins [116,148]. Wild type mice fed a high-fat diet develop obesity, hyperlipidemia, type 2 diabetes mellitus, insulin resistance, and nonalcoholic steatohepatitis [149–151]. These effects of high fat feeding are significantly accelerated in mice lacking SIRT3 [148]. Chronic HFD reduces SIRT3 mRNA and protein levels in the liver leading to hyperacetylation of mitochondrial proteins [148,152]. The suppression of SIRT3 occurs at the transcriptional level and is primarily driven by the HFD-induced suppression of PGC-1α, a major regulator of *SIRT3* expression [153]. Overexpression of exogenous *PGC-1α* is sufficient to rescue the loss of *SIRT3* in HFD-fed mice [154]. A similar finding is found in the muscle [155]. HFD feeding is also associated with increased fatty-acid oxidation and increased mitochondrial acetyl-CoA levels, and *SIRT3* expression is induced early after initiation of high-fat feeding [148]. However, in contrast to caloric restriction or fasting-induced S*IRT3* expression, chronic HFD feeding suppresses *SIRT3* expression, increases mitochondrial protein acetylation, and ultimately reduces fatty-acid oxidation. Interestingly, mice lacking *SIRT3* under HFD show accelerated characteristics of the metabolic syndrome such as obesity, insulin resistance, hyperlipidemia and steatohepatitis compared to WT [148].

SIRT3 also modulates oxygen consumption and ROS levels in hepatocytes [156]. SIRT3 regulates the antioxidant capacity of the cell by directly modulating key antioxidant enzymes which act to prevent oxidative damage. SIRT3 antioxidant effects are mediated in part by its interaction with MnSOD and isocitrate dehydrogenase 2 (IDH2). SIRT3 deacetylates and activates isocitrate deshydrogenase 2 (IDH2), an enzyme of the tricarboxylic acid cycle which produces NADPH [157–159]. SIRT3 also controls the acetylation and the activity of superoxide dismutase 2 (SOD2), the primary mitochondrial antioxidant enzyme that converts O^−2^ to H_2_O_2_, which is further converted to water [160]. SIRT3 directly deacetylates SOD2 in mitochondria and enhances its ability to scavenge ROS [147,161]. SIRT3^−/−^ mice exhibit decreased SOD2 activity and increased oxidative stress suggesting a role of SIRT3 in NASH [147,161].

*SIRT3*^−/−^ mice subjected to methionine choline deficient diet (MCD) exhibit increased serum ALT levels, increased hepatic content, higher expression of inflammatory and fibrogenic genes and reduced (SOD2) activity. Conversely, overexpression of *SIRT3* results in opposite effects suggesting that *SIRT3* ablation aggravates MCD induced NASH while *SIRT3* overexpression alleviates the MCD induced phenotype [162].

### 4.3. Molecular Pathways Regulating Mitochondrial Content

Mitochondrial biogenesis and mitochondrial specific autophagy (mitophagy) are two pathways that regulate mitochondrial mass. Imbalance between mitochondrial biogenesis and mitophagy is a highly regulated process that influences both mitochondrial and cellular homeostasis. Increasing evidence suggests that mitochondrial biogenesis and autophagy play an important role in lipid metabolism, insulin resistance and may therefore play a role in NAFLD [163–165]. Several studies involve autophagy in hepatic steatosis, however the role of specific autophagies such as mitophagy remains unclear [163–165].

Mitochondrial biogenesis is a multistep process, requiring the coordinated transcription and translation of both mitochondrial and nuclear-originated transcripts and recruitment of newly synthesized proteins and lipids. The known regulators of mitochondrial biogenesis are PGC-1α, adenosine monophosphate (AMP)-activated protein kinase (AMPK), SIRT1 and SIRT3, endothelial nitric oxide synthase (eNOS), NRFs, mitochondrial transcription factor A (TFAM). PGC-1α plays a crucial role in the regulation of mitochondrial biogenesis by coordinating the activity NRFs and ERRs [166]. NRF1 and NRF2 regulate the transcription of mitochondrial transcription factor A (TFAM) and transcription factor B proteins (TFBs) which are major regulators of mitochondrial DNA transcription and replication [167]. ERRs is also a downstream target for *PGC-1α. ERR-α* is known to regulate the transcription of nuclear genes encoding mitochondria-related factors, including those involved in oxidative phosphorylation, fatty acid oxidation, Kreb’s cycle and mitochondrial fission and fusion [35,166]. Moreover, expression of *PGC-1α* or *PGC-1β* in human cells deficient of complex III or IV showed that expression of *PGC-1α* or *PGC-1β* improves mitochondrial respiration suggesting a major role for PGC-1α in mitochondrial biogenesis and function [168]. PGC-1α itself is the subject of modulations through metabolic sensors such as AMPK, which senses the cellular energy demands, and p38, which senses fluctuations in cytoplasmic calcium levels. AMPK also promotes SIRT1 activity by increasing cellular NAD^+^ levels. SIRT1 deacetylates PGC-1α and promotes mitochondrial content and oxidative metabolism. In addition, cytosolic calcium concentration affects mitochondrial biogenesis through the activation of p38 mitogen-activated kinase and calcium/calmodulin-dependent kinase (CaMK) which in turn modulate PGC-1α activity [119,120]. The increase in *SIRT3* expression is important for PGC-1α mediated induction of mitochondrial biogenesis [169,170]. *SIRT3* deletion inhibits PGC-1α-induced mitochondrial biogenesis [169]. PGC-1α is an upstream activator of SIRT3; deletion of *PGC-1α* reduces the expression of *SIRT3* and reduces mitochondrial biogenesis [169,171].

Mitophagy is the selective removal of damaged mitochondria by autophagosomes and their subsequent catabolism by lysosomes [172]. Mitophagy is crucial for the maintenance of mitochondrial content and integrity. Mitochondria undergo fission before mitophagy, supporting the separation of healthy mitochondria from dysfunctional ones [173–175]. The most well-studied components of fission machinery are dynamin related protein-1 (Drp1) and fission protein 1 (Fis1) [173]. Mitophagy is triggered by loss of mitochondrial membrane potential. Various proteins have been implicated in the regulation of mitophagy including the phosphatase and tensin homolog-induced putative kinase 1 (PINK1) and Parkin. The serine/threonine kinase PINK1 plays a central role in communicating the loss of membrane potential to Parkin [176]. It has been shown that the recruitment of Parkin to impaired mitochondria requires *PINK1* expression and its kinase activity [177,178]. The E3 ubiquitin ligase Parkin is predominantly cytosolic under basal conditions but rapidly translocates to mitochondria upon loss of mitochondrial membrane potential. Parkin promotes ubiquitination of proteins in the mitochondrial membrane which triggers the degradation of the damaged mitochondria by autophagosomes and lysosomes. The substrates identified for parkin are voltage-dependent anion channel 1 (VDAC1), mitofusin-1 and mitofusin-2 (MFN1/2) and the mitochondrial Rho GTPases (Miro) [177,179,180]. Parkin is proposed to prevent mitochondrial fusion through mediating the degradation of MFN1 and MFN2, thereby segregating depolarized mitochondria from healthy mitochondria [180,181].

## 5. Prevention and Treatment of NAFLD

Current therapies to improve NASH include life style modification through caloric restriction and/or exercise [182–190]. Exercise has been shown to improve insulin resistance and liver histology in NAFLD patients [182]. Physical activity has been shown to improve mitochondrial function in patients with NAFLD [185]. Endurance exercise improved the impaired bioenergetics and the structural damage induced by NASH [186]. Studies from our group have shown that exercise increases the activity of mitochondrial fatty acid oxidation and prevents hepatic fat accumulation [190]. Moreover, rats bred for high aerobic capacity have higher oxidative capacity and reduced hepatic fat [87].

The current pharmacological therapies used for the treatment of NASH include the use of antioxidants such vitamin E and insulin sensitizers. Vitamin E is present at low levels in patients with NASH and is recommended for treatment of NASH in the absence of diabetes or cirrhosis; however some studies linked vitamin E to prostate cancer and hemorrhagic stroke [191]. Insulin sensitizers used as a therapy for NASH include thiazolidinedione (TZDS) and metformin. Pioglitazone is a thiazolidinedione that has been shown to improve insulin sensitivity through activation of the nuclear peroxisome proliferator activated receptor gamma which is involved in the regulation of many genes involved in glucose and lipid metabolism. Pioglitazone treatment is associated with improvement of insulin resistance and liver histology but was also associated with weight gain. Metformin, a biguanide used to treat type 2 diabetes, has been shown to improve insulin sensitivity, and liver TG content but requires long term treatment for the prevention of the disease and was associated with weight loss. In a recent study from our group using obese type-2 diabetic rats, aerobic exercise training was more effective (through improvement of mitochondrial function) than metformin in the management of NAFLD and type 2 diabetes [190,192]. Combining metformin and exercise had no significant improvement compared to exercise alone [192]. Treatment with resveratrol, a polyphenol found in wine and grapes, has been shown to mimic caloric restriction and prevent high fat diet induced steatosis through regulation of key metabolic regulators of energy: SIRT1 and AMPK. The therapeutic efficacy of resveratrol in clinical NAFLD is not yet established due to inconsistent data and variability in the dosage, the health of the patients, and the duration of the treatment [132,193–196]. As mentioned above, rats fed high fat diets have impaired liver bioenergetics. Berberine, an isoquinoline alkaloid with anti-diabetic properties, improved the effect of a high fat diet. The effect of berberine is associated with increased SIRT3 levels and activity in the mitochondria [197]. More controlled studies in animals and humans are required to determine the efficacy of sirtuins in the treatment of NAFLD.

## 6. Conclusions

NAFLD has become a world-wide epidemic aggravated by the consistent increase in obesity over the recent years. The development of advanced forms of NAFLD implicates factors such as FFAs, cytokines, adipokines, mitochondrial dysfunction and oxidative stress. The interplay of these factors in the pathogenesis of NAFLD in not completely understood. Recent findings on mechanisms regulating mitochondrial content and function offer new potential therapeutic avenues for the treatment of NAFLD.

## Figures and Tables

**Table 1.** Selected published reports on the role of mitochondria in NAFLD.

Authors	Journal	Title
Sanyal *et al.* [78]	*Gastroenterology* **2001**, *120*, 1183–1192	Nonalcoholic steatohepatitis: association of insulin resistance and mitochondrial abnormalities
Ibdah *et al.* [84]	*Gastroenterology* **2005**, *128*, 1381–1390	Mice heterozygous for a defect in mitochondrial trifunctional protein develop hepatic steatosis and insulin resistance
Zhou *et al.* [86]	*Hepatology* **2008**, *48*, 1087–1096	Mitochondrial dysfunction contributes to the increased vulnerabilities of adiponectin knockout mice to liver injury
Thyfault *et al.* [87]	*J. Physiol.* **2009**, *587*, 1805–1816	Rats selectively bred for low aerobic capacity have reduced hepatic mitochondrial oxidative capacity and susceptibility to hepatic steatosis and injury
Rector *et al.* [88]	*J. Hepatol.* **2010**, *52*, 727–736	Mitochondrial dysfunction precedes insulin resistance and hepatic steatosis and contributes to the natural history of non-alcoholic fatty liver disease in an obese rodent model
Rector *et al.* [85]	*Hepatology* **2013**, *57*, 2213–2223	Selective hepatic insulin resistance in a murine model heterozygous for a mitochondrial trifunctional protein defect

## References

[1] Milic S., Stimac D. (2012). Nonalcoholic fatty liver disease/steatohepatitis: Epidemiology, pathogenesis, clinical presentation and treatment. Dig. Dis. (Basel. Switz.).

[2] Nassir F., Adewole O.L., Brunt E.M., Abumrad N.A. (2013). CD36 deletion reduces VLDL secretion, modulates liver prostaglandins, and exacerbates hepatic steatosis in ob/ob mice. J. Lipid Res.

[3] Cohen J.C., Horton J.D., Hobbs H.H. (2011). Human fatty liver disease: Old questions and new insights. Science.

[4] Fabbrini E., Sullivan S., Klein S. (2010). Obesity and nonalcoholic fatty liver disease: Biochemical, metabolic, and clinical implications. Hepatology (Baltim., Md.).

[5] Grattagliano I., Russmann S., Diogo C., Bonfrate L., Oliveira P.J., Wang D.Q., Portincasa P. (2011). Mitochondria in chronic liver disease. Curr. Drug Targets.

[6] Szczepaniak L.S., Nurenberg P., Leonard D., Browning J.D., Reingold J.S., Grundy S., Hobbs H.H., Dobbins R.L. (2005). Magnetic resonance spectroscopy to measure hepatic triglyceride content: Prevalence of hepatic steatosis in the general population. Am. J. Physiol. Endocrinol. Metab.

[7] Scaglioni F., Ciccia S., Marino M., Bedogni G., Bellentani S. (2011). ASH and NASH. Dig. Dis. (Basel, Switz.).

[8] Chalasani N., Younossi Z., Lavine J.E., Diehl A.M., Brunt E.M., Cusi K., Charlton M., Sanyal A.J. (2012). The diagnosis and management of non-alcoholic fatty liver disease: Practice Guideline by the American Association for the Study of Liver Diseases, American College of Gastroenterology, and the American Gastroenterological Association. Hepatology (Baltim., Md.).

[9] Adams L.A., Lymp J.F., St Sauver J., Sanderson S.O., Lindor K.D., Feldstein A., Angulo P. (2005). The natural history of nonalcoholic fatty liver disease: A population-based cohort study. Gastroenterology.

[10] Vernon G., Baranova A., Younossi Z.M. (2011). Systematic review: The epidemiology and natural history of non-alcoholic fatty liver disease and non-alcoholic steatohepatitis in adults. Aliment. Pharmacol. Therap.

[11] Tetri L.H., Basaranoglu M., Brunt E.M., Yerian L.M., Neuschwander-Tetri B.A. (2008). Severe NAFLD with hepatic necroinflammatory changes in mice fed trans fats and a high-fructose corn syrup equivalent. Am. J. Physiol. Gastrointest. Liver Physiol.

[12] Alisi A., Manco M., Panera N., Nobili V. (2009). Association between type two diabetes and non-alcoholic fatty liver disease in youth. Ann. Hepatol.

[13] Parikh R.M., Mohan V. (2012). Changing definitions of metabolic syndrome. Indian J. Endocrinol. Metab.

[14] Rahman R., Hammoud G.M., Almashhrawi A.A., Ahmed K.T., Ibdah J.A. (2013). Primary hepatocellular carcinoma and metabolic syndrome: An update. World J. Gastrointest. Oncol.

[15] Clark J.M., Brancati F.L., Diehl A.M. (2002). Nonalcoholic fatty liver disease. Gastroenterology.

[16] Machado M., Marques-Vidal P., Cortez-Pinto H. (2006). Hepatic histology in obese patients undergoing bariatric surgery. J. Hepatol.

[17] Loria P., Lonardo A., Anania F. (2013). Liver and diabetes: A vicious circle. Hepatol. Res.

[18] Takamura T., Misu H., Ota T., Kaneko S. (2012). Fatty liver as a consequence and cause of insulin resistance: Lessons from type 2 diabetic liver. Endocr. J.

[19] Younossi Z.M., Gramlich T., Matteoni C.A., Boparai N., McCullough A.J. (2004). Nonalcoholic fatty liver disease in patients with type 2 diabetes. Clin. Gastroenterol. Hepatol.

[20] Abdelmalek M., Ludwig J., Lindor K.D. (1995). Two cases from the spectrum of nonalcoholic steatohepatitis. J. Clin. Gastroenterol.

[21] Bacon B.R., Farahvash M.J., Janney C.G., Neuschwander-Tetri B.A. (1994). Nonalcoholic steatohepatitis: An expanded clinical entity. Gastroenterology.

[22] Teli M.R., James O.F., Burt A.D., Bennett M.K., Day C.P. (1995). The natural history of nonalcoholic fatty liver: A follow-up study. Hepatology (Baltim., Md.).

[23] Guerrero R., Vega G.L., Grundy S.M., Browning J.D. (2009). Ethnic differences in hepatic steatosis: An insulin resistance paradox?. Hepatology (Baltim., Md.).

[24] Lewis J.R., Mohanty S.R. (2010). Nonalcoholic fatty liver disease: A review and update. Dig. Dis. Sci.

[25] Day C.P., James O.F. (1998). Steatohepatitis: A tale of two “hits”?. Gastroenterology.

[26] Tilg H., Moschen A.R. (2010). Evolution of inflammation in nonalcoholic fatty liver disease: The multiple parallel hits hypothesis. Hepatology (Baltim., Md.).

[27] Lonardo A., Bellentani S., Ratziu V., Loria P. (2011). Insulin resistance in nonalcoholic steatohepatitis: Necessary but not sufficient—Death of a dogma from analysis of therapeutic studies?. Expert Rev. Gastroenterol. Hepatol.

[28] Yilmaz Y. (2012). Review article: Is non-alcoholic fatty liver disease a spectrum, or are steatosis and non-alcoholic steatohepatitis distinct conditions?. Aliment. Pharmacol. Therap.

[29] Amaro A., Fabbrini E., Kars M., Yue P., Schechtman K., Schonfeld G., Klein S. (2010). Dissociation between intrahepatic triglyceride content and insulin resistance in familial hypobetalipoproteinemia. Gastroenterology.

[30] Kantartzis K., Machicao F., Machann J., Schick F., Fritsche A., Haring H.U., Stefan N. (2009). The *DGAT2* gene is a candidate for the dissociation between fatty liver and insulin resistance in humans. Clin. Sci. (Lond., Engl.).

[31] Kantartzis K., Peter A., Machicao F., Machann J., Wagner S., Konigsrainer I., Konigsrainer A., Schick F., Fritsche A., Haring H.U. (2009). Dissociation between fatty liver and insulin resistance in humans carrying a variant of the patatin-like phospholipase 3 gene. Diabetes.

[32] Stefan N., Staiger H., Haring H.U. (2011). Dissociation between fatty liver and insulin resistance: The role of adipose triacylglycerol lipase. Diabetologia.

[33] Brumbaugh D.E., Friedman J.E. (2014). Developmental origins of nonalcoholic fatty liver disease. Pediatr. Res.

[34] Nestel P.J., Havel R.J., Bezman A. (1962). Sites of initial removal of chylomicron triglyceride fatty acids from the blood. J. Clin. Investig.

[35] Donnelly K.L., Smith C.I., Schwarzenberg S.J., Jessurun J., Boldt M.D., Parks E.J. (2005). Sources of fatty acids stored in liver and secreted via lipoproteins in patients with nonalcoholic fatty liver disease. J. Clin. Investig.

[36] Lafontan M., Langin D. (2009). Lipolysis and lipid mobilization in human adipose tissue. Prog. Lipid Res.

[37] Ferre P., Foufelle F. (2010). Hepatic steatosis: A role for *de novo* lipogenesis and the transcription factor SREBP-1c. Diabetes Obes. Metab.

[38] Horton J.D., Goldstein J.L., Brown M.S. (2002). SREBPs: Transcriptional mediators of lipid homeostasis. Cold Spring Harb. Symp. Quant. Biol.

[39] Kazantzis M., Stahl A. (2012). Fatty acid transport proteins, implications in physiology and disease. Biochim. Biophys. Acta.

[40] Doege H., Baillie R.A., Ortegon A.M., Tsang B., Wu Q., Punreddy S., Hirsch D., Watson N., Gimeno R.E., Stahl A. (2006). Targeted deletion of FATP5 reveals multiple functions in liver metabolism: Alterations in hepatic lipid homeostasis. Gastroenterology.

[41] Doege H., Grimm D., Falcon A., Tsang B., Storm T.A., Xu H., Ortegon A.M., Kazantzis M., Kay M.A., Stahl A. (2008). Silencing of hepatic fatty acid transporter protein 5 *in vivo* reverses diet-induced non-alcoholic fatty liver disease and improves hyperglycemia. J. Biol. Chem.

[42] Falcon A., Doege H., Fluitt A., Tsang B., Watson N., Kay M.A., Stahl A. (2010). FATP2 is a hepatic fatty acid transporter and peroxisomal very long-chain acyl-CoA synthetase. Am. J. Physiol. Endocrinol. Metab.

[43] Fernandez M.A., Albor C., Ingelmo-Torres M., Nixon S.J., Ferguson C., Kurzchalia T., Tebar F., Enrich C., Parton R.G., Pol A. (2006). Caveolin-1 is essential for liver regeneration. Science.

[44] Su X., Abumrad N.A. (2009). Cellular fatty acid uptake: A pathway under construction. Trends Endocrinol. Metab.

[45] Koonen D.P.Y., Jacobs R.L., Febbraio M., Young M.E., Soltys C.-L.M., Ong H., Vance D.E., Dyck J.R.B. (2007). Increased hepatic CD36 expression contributes to dyslipidemia associated with diet-induced obesity. Diabetes.

[46] Miquilena-Colina M.E., Lima-Cabello E., Sanchez-Campos S., Garcia-Mediavilla M.V., Fernandez-Bermejo M., Lozano-Rodriguez T., Vargas-Castrillon J., Buque X., Ochoa B., Aspichueta P. (2011). Hepatic fatty acid translocase CD36 upregulation is associated with insulin resistance, hyperinsulinaemia and increased steatosis in non-alcoholic steatohepatitis and chronic hepatitis, C. Gut.

[47] Zhou D., Samovski D., Okunade A.L., Stahl P.D., Abumrad N.A., Su X. (2012). CD36 level and trafficking are determinants of lipolysis in adipocytes. FASEB J.

[48] Fabbrini E., Magkos F., Mohammed B.S., Pietka T., Abumrad N.A., Patterson B.W., Okunade A., Klein S. (2009). Intrahepatic fat, not visceral fat, is linked with metabolic complications of obesity. Proc. Natl. Acad. Sci. USA.

[49] Stremmel W., Pohl L., Ring A., Herrmann T. (2001). A new concept of cellular uptake and intracellular trafficking of long-chain fatty acids. Lipids.

[50] Storch J., Corsico B. (2008). The emerging functions and mechanisms of mammalian fatty acid-binding proteins. Annu. Rev. Nutr.

[51] Newberry E.P., Xie Y., Kennedy S., Han X., Buhman K.K., Luo J., Gross R.W., Davidson N.O. (2003). Decreased hepatic triglyceride accumulation and altered fatty acid uptake in mice with deletion of the liver fatty acid-binding protein gene. J. Biol. Chem.

[52] Wolins N.E., Brasaemle D.L., Bickel P.E. (2006). A proposed model of fat packaging by exchangeable lipid droplet proteins. FEBS Lett.

[53] Schonfeld G. (2003). Familial hypobetalipoproteinemia: A review. J. Lipid Res.

[54] Sen D., Dagdelen S., Erbas T. (2007). Hepatosteatosis with hypobetalipoproteinemia. J. Natl. Med. Assoc.

[55] Ginsberg H.N., Fisher E.A. (2009). The ever-expanding role of degradation in the regulation of apolipoprotein B metabolism. J. Lipid Res.

[56] Tarugi P., Lonardo A., Ballarini G., Grisendi A., Pulvirenti M., Bagni A., Calandra S. (1996). Fatty liver in heterozygous hypobetalipoproteinemia caused by a novel truncated form of apolipoprotein, B. Gastroenterology.

[57] Welty F.K. (2014). Hypobetalipoproteinemia and abetalipoproteinemia. Curr. Opin. Lipidol.

[58] Wolfrum C., Stoffel M. (2006). Coactivation of Foxa2 through Pgc-1β promotes liver fatty acid oxidation and triglyceride/VLDL secretion. Cell Metab.

[59] Choi S.H., Ginsberg H.N. (2011). Increased very low density lipoprotein (VLDL) secretion, hepatic steatosis, and insulin resistance. Trends Endocrinol. Metab.

[60] Van der Poorten D., Samer C.F., Ramezani-Moghadam M., Coulter S., Kacevska M., Schrijnders D., Wu L.E., McLeod D., Bugianesi E., Komuta M. (2013). Hepatic fat loss in advanced nonalcoholic steatohepatitis: Are alterations in serum adiponectin the cause?. Hepatology (Baltim., Md.).

[61] Begriche K., Massart J., Robin M.A., Bonnet F., Fromenty B. (2013). Mitochondrial adaptations and dysfunctions in nonalcoholic fatty liver disease. Hepatology (Baltim., Md.).

[62] Eaton S., Bartlett K., Pourfarzam M. (1996). Mammalian mitochondrial β-oxidation. Biochem. J.

[63] McGarry J.D., Foster D.W. (1980). Regulation of hepatic fatty acid oxidation and ketone body production. Annu. Rev. Biochem.

[64] Sidossis L.S., Stuart C.A., Shulman G.I., Lopaschuk G.D., Wolfe R.R. (1996). Glucose plus insulin regulate fat oxidation by controlling the rate of fatty acid entry into the mitochondria. J. Clin. Investig.

[65] Longuet C., Sinclair E.M., Maida A., Baggio L.L., Maziarz M., Charron M.J., Drucker D.J. (2008). The glucagon receptor is required for the adaptive metabolic response to fasting. Cell Metab.

[66] Mandard S., Muller M., Kersten S. (2004). Peroxisome proliferator-activated receptor α target genes. Cell. Mol. Life Sci.

[67] Potthoff M.J., Inagaki T., Satapati S., Ding X., He T., Goetz R., Mohammadi M., Finck B.N., Mangelsdorf D.J., Kliewer S.A. (2009). FGF21 induces PGC-1α and regulates carbohydrate and fatty acid metabolism during the adaptive starvation response. Proc. Natl. Acad. Sci. USA.

[68] Pellicoro A., Ramachandran P., Iredale J.P., Fallowfield J.A. (2014). Liver fibrosis and repair: Immune regulation of wound healing in a solid organ. Nat. Rev. Immunol.

[69] Wang K. (2014). Molecular mechanisms of hepatic apoptosis. Cell Death Dis.

[70] Malaguarnera M., di Rosa M., Nicoletti F., Malaguarnera L. (2009). Molecular mechanisms involved in NAFLD progression. J. Mol. Med. (Berl., Ger.).

[71] Musso G., Gambino R., Cassader M. (2010). Obesity, diabetes, and gut microbiota: The hygiene hypothesis expanded?. Diabetes Care.

[72] Calvo S.E., Mootha V.K. (2010). The mitochondrial proteome and human disease. Annu. Rev. Genomics Hum. Genet.

[73] Pessayre D., Mansouri A., Fromenty B. (2002). Nonalcoholic steatosis and steatohepatitis. V. Mitochondrial dysfunction in steatohepatitis. Am. J. Physiol. Gastrointest. Liver Physiol.

[74] Boveris A., Chance B. (1973). The mitochondrial generation of hydrogen peroxide. General properties and effect of hyperbaric oxygen. Biochem. J.

[75] Begriche K., Igoudjil A., Pessayre D., Fromenty B. (2006). Mitochondrial dysfunction in NASH: Causes, consequences and possible means to prevent it. Mitochondrion.

[76] Mansouri A., Gaou I., de Kerguenec C., Amsellem S., Haouzi D., Berson A., Moreau A., Feldmann G., Letteron P., Pessayre D. (1999). An alcoholic binge causes massive degradation of hepatic mitochondrial DNA in mice. Gastroenterology.

[77] Caldwell S.H., Chang C.Y., Nakamoto R.K., Krugner-Higby L. (2004). Mitochondria in nonalcoholic fatty liver disease. Clin. Liver Dis.

[78] Sanyal A.J., Campbell-Sargent C., Mirshahi F., Rizzo W.B., Contos M.J., Sterling R.K., Luketic V.A., Shiffman M.L., Clore J.N. (2001). Nonalcoholic steatohepatitis: Association of insulin resistance and mitochondrial abnormalities. Gastroenterology.

[79] Lammens M., Laak H. (2005). Contribution of histopathological examination to the diagnosis of OXPHOS disorders. Oxidative Phosphorylation in Health and Disease.

[80] Rolo A.P., Teodoro J.S., Palmeira C.M. (2012). Role of oxidative stress in the pathogenesis of nonalcoholic steatohepatitis. Free Radic. Biol. Med.

[81] Ibdah J.A., Paul H., Zhao Y., Binford S., Salleng K., Cline M., Matern D., Bennett M.J., Rinaldo P., Strauss A.W. (2001). Lack of mitochondrial trifunctional protein in mice causes neonatal hypoglycemia and sudden death. J. Clin. Investig.

[82] Wanders R.J., Ijist L., Poggi F., Bonnefont J.P., Munnich A., Brivet M., Rabier D., Saudubray J.M. (1992). Human trifunctional protein deficiency: A new disorder of mitochondrial fatty acid beta-oxidation. Biochem. Biophys. Res. Commun.

[83] Rector R.S., Payne R.M., Ibdah J.A. (2008). Mitochondrial trifunctional protein defects: Clinical implications and therapeutic approaches. Adv. Drug Deliv. Rev.

[84] Ibdah J.A., Perlegas P., Zhao Y., Angdisen J., Borgerink H., Shadoan M.K., Wagner J.D., Matern D., Rinaldo P., Cline J.M. (2005). Mice heterozygous for a defect in mitochondrial trifunctional protein develop hepatic steatosis and insulin resistance. Gastroenterology.

[85] Rector R.S., Morris E.M., Ridenhour S., Meers G.M., Hsu F.F., Turk J., Ibdah J.A. (2013). Selective hepatic insulin resistance in a murine model heterozygous for a mitochondrial trifunctional protein defect. Hepatology.

[86] Zhou M., Xu A., Tam P.K., Lam K.S., Chan L., Hoo R.L., Liu J., Chow K.H., Wang Y. (2008). Mitochondrial dysfunction contributes to the increased vulnerabilities of adiponectin knockout mice to liver injury. Hepatology.

[87] Thyfault J.P., Rector R.S., Uptergrove G.M., Borengasser S.J., Morris E.M., Wei Y., Laye M.J., Burant C.F., Qi N.R., Ridenhour S.E. (2009). Rats selectively bred for low aerobic capacity have reduced hepatic mitochondrial oxidative capacity and susceptibility to hepatic steatosis and injury. J. Physiol.

[88] Rector R.S., Thyfault J.P., Uptergrove G.M., Morris E.M., Naples S.P., Borengasser S.J., Mikus C.R., Laye M.J., Laughlin M.H., Booth F.W. (2010). Mitochondrial dysfunction precedes insulin resistance and hepatic steatosis and contributes to the natural history of non-alcoholic fatty liver disease in an obese rodent model. J. Hepatol.

[89] Kawano K., Hirashima T., Mori S., Saitoh Y., Kurosumi M., Natori T. (1992). Spontaneous long-term hyperglycemic rat with diabetic complications. Otsuka long-evans tokushima fatty (OLETF) strain. Diabetes.

[90] Jeong S.K., Kim Y.K., Park J.W., Shin Y.J., Kim D.S. (2008). Impact of visceral fat on the metabolic syndrome and nonalcoholic fatty liver disease. J. Korean Med. Sci.

[91] Wang Y., Zhou M., Lam K.S., Xu A. (2009). Protective roles of adiponectin in obesity-related fatty liver diseases: Mechanisms and therapeutic implications. Arq. Bras. Endocrinol. Metabol.

[92] Bajaj M., Suraamornkul S., Piper P., Hardies L.J., Glass L., Cersosimo E., Pratipanawatr T., Miyazaki Y., DeFronzo R.A. (2004). Decreased plasma adiponectin concentrations are closely related to hepatic fat content and hepatic insulin resistance in pioglitazone-treated type 2 diabetic patients. J. Clin. Endocrinol. Metab.

[93] Hui J.M., Hodge A., Farrell G.C., Kench J.G., Kriketos A., George J. (2004). Beyond insulin resistance in NASH: TNF-α or adiponectin?. Hepatology (Baltim. Md.).

[94] Jung U.J., Choi M.S. (2014). Obesity and its metabolic complications: The role of adipokines and the relationship between obesity, inflammation, insulin resistance, dyslipidemia and nonalcoholic fatty liver disease. Int. J. Mol. Sci.

[95] Negre-Salvayre A., Hirtz C., Carrera G., Cazenave R., Troly M., Salvayre R., Penicaud L., Casteilla L. (1997). A role for uncoupling protein-2 as a regulator of mitochondrial hydrogen peroxide generation. FASEB J.

[96] Chow L., From A., Seaquist E. (2010). Skeletal muscle insulin resistance: The interplay of local lipid excess and mitochondrial dysfunction. Metab. Clin. Exp.

[97] Morino K., Petersen K.F., Dufour S., Befroy D., Frattini J., Shatzkes N., Neschen S., White M.F., Bilz S., Sono S. (2005). Reduced mitochondrial density and increased IRS-1 serine phosphorylation in muscle of insulin-resistant offspring of type 2 diabetic parents. J. Clin. Investig.

[98] Petersen K.F., Dufour S., Befroy D., Garcia R., Shulman G.I. (2004). Impaired mitochondrial activity in the insulin-resistant offspring of patients with type 2 diabetes. N. Engl. J. Med.

[99] Morino K., Petersen K.F., Sono S., Choi C.S., Samuel V.T., Lin A., Gallo A., Zhao H., Kashiwagi A., Goldberg I.J. (2012). Regulation of mitochondrial biogenesis by lipoprotein lipase in muscle of insulin-resistant offspring of parents with type 2 diabetes. Diabetes.

[100] Petersen K.F., Dufour S., Morino K., Yoo P.S., Cline G.W., Shulman G.I. (2012). Reversal of muscle insulin resistance by weight reduction in young, lean, insulin-resistant offspring of parents with type 2 diabetes. Proc. Natl. Acad. Sci. USA.

[101] Andersson U., Scarpulla R.C. (2001). Pgc-1-related coactivator, a novel, serum-inducible coactivator of nuclear respiratory factor 1-dependent transcription in mammalian cells. Mol. Cell. Biol.

[102] Vercauteren K., Gleyzer N., Scarpulla R.C. (2009). Short hairpin RNA-mediated silencing of PRC (PGC-1-related coactivator) results in a severe respiratory chain deficiency associated with the proliferation of aberrant mitochondria. J. Biol. Chem.

[103] Handschin C., Spiegelman B.M. (2006). Peroxisome proliferator-activated receptor gamma coactivator 1 coactivators, energy homeostasis, and metabolism. Endocr. Rev.

[104] Lin J., Puigserver P., Donovan J., Tarr P., Spiegelman B.M. (2002). Peroxisome proliferator-activated receptor γ coactivator 1β (PGC-1β ), a novel PGC-1-related transcription coactivator associated with host cell factor. J. Biol. Chem.

[105] Puigserver P., Wu Z., Park C.W., Graves R., Wright M., Spiegelman B.M. (1998). A cold-inducible coactivator of nuclear receptors linked to adaptive thermogenesis. Cell.

[106] Lai L., Leone T.C., Zechner C., Schaeffer P.J., Kelly S.M., Flanagan D.P., Medeiros D.M., Kovacs A., Kelly D.P. (2008). Transcriptional coactivators PGC-1α and PGC-lβ control overlapping programs required for perinatal maturation of the heart. Genes Dev.

[107] Lelliott C.J., Medina-Gomez G., Petrovic N., Kis A., Feldmann H.M., Bjursell M., Parker N., Curtis K., Campbell M., Hu P. (2006). Ablation of PGC-1β results in defective mitochondrial activity, thermogenesis, hepatic function, and cardiac performance. PLoS Biol.

[108] Leone T.C., Lehman J.J., Finck B.N., Schaeffer P.J., Wende A.R., Boudina S., Courtois M., Wozniak D.F., Sambandam N., Bernal-Mizrachi C. (2005). PGC-1α deficiency causes multi-system energy metabolic derangements: Muscle dysfunction, abnormal weight control and hepatic steatosis. PLoS Biol.

[109] Sonoda J., Mehl I.R., Chong L.W., Nofsinger R.R., Evans R.M. (2007). PGC-1β controls mitochondrial metabolism to modulate circadian activity, adaptive thermogenesis, and hepatic steatosis. Proc. Natl. Acad. Sci. USA.

[110] Vianna C.R., Huntgeburth M., Coppari R., Choi C.S., Lin J., Krauss S., Barbatelli G., Tzameli I., Kim Y.B., Cinti S. (2006). Hypomorphic mutation of PGC-1β causes mitochondrial dysfunction and liver insulin resistance. Cell Metab.

[111] Horton J.D., Shah N.A., Warrington J.A., Anderson N.N., Park S.W., Brown M.S., Goldstein J.L. (2003). Combined analysis of oligonucleotide microarray data from transgenic and knockout mice identifies direct SREBP target genes. Proc. Natl. Acad. Sci. USA.

[112] Lin J., Yang R., Tarr P.T., Wu P.-H., Handschin C., Li S., Yang W., Pei L., Uldry M., Tontonoz P. (2005). Hyperlipidemic effects of dietary saturated fats mediated through PGC-1β coactivation of SREBP. Cell.

[113] Bellafante E., Murzilli S., Salvatore L., Latorre D., Villani G., Moschetta A. (2013). Hepatic-specific activation of peroxisome proliferator-activated receptor γ coactivator-1β protects against steatohepatitis. Hepatology (Baltim. Md.).

[114] Morris E.M., Meers G.M., Booth F.W., Fritsche K.L., Hardin C.D., Thyfault J.P., Ibdah J.A. (2012). PGC-1α overexpression results in increased hepatic fatty acid oxidation with reduced triacylglycerol accumulation and secretion. Am. J. Physiol. Gastrointest. Liver Physiol.

[115] Laye M.J., Rector R.S., Borengasser S.J., Naples S.P., Uptergrove G.M., Ibdah J.A., Booth F.W., Thyfault J.P. (2009). Cessation of daily wheel running differentially alters fat oxidation capacity in liver, muscle, and adipose tissue. J. Appl. Physiol. (Bethesda. Md.).

[116] Hirschey M.D., Shimazu T., Jing E., Grueter C.A., Collins A.M., Aouizerat B., Stancakova A., Goetzman E., Lam M.M., Schwer B. (2011). SIRT3 deficiency and mitochondrial protein hyperacetylation accelerate the development of the metabolic syndrome. Mol. Cell.

[117] Puigserver P. (2005). Tissue-specific regulation of metabolic pathways through the transcriptional coactivator PGC1-α. Int. J. Obes. Relat. Metab. Disord.

[118] Wu Z., Puigserver P., Andersson U., Zhang C., Adelmant G., Mootha V., Troy A., Cinti S., Lowell B., Scarpulla R.C. (1999). Mechanisms controlling mitochondrial biogenesis and respiration through the thermogenic coactivator PGC-1. Cell.

[119] Feige J.N., Auwerx J. (2007). Transcriptional coregulators in the control of energy homeostasis. Trends Cell Biol.

[120] Wright D.C., Geiger P.C., Han D.H., Jones T.E., Holloszy J.O. (2007). Calcium induces increases in peroxisome proliferator-activated receptor γ coactivator-1α and mitochondrial biogenesis by a pathway leading to p38 mitogen-activated protein kinase activation. J. Biol. Chem.

[121] Canto C., Gerhart-Hines Z., Feige J.N., Lagouge M., Noriega L., Milne J.C., Elliott P.J., Puigserver P., Auwerx J. (2009). AMPK regulates energy expenditure by modulating NAD^+^ metabolism and SIRT1 activity. Nature.

[122] Haigis M.C., Guarente L.P. (2006). Mammalian sirtuins—Emerging roles in physiology, aging, and calorie restriction. Genes Dev.

[123] Haigis M.C., Sinclair D.A. (2010). Mammalian sirtuins: Biological insights and disease relevance. Annu. Rev. Pathol.

[124] Nogueiras R., Habegger K.M., Chaudhary N., Finan B., Banks A.S., Dietrich M.O., Horvath T.L., Sinclair D.A., Pfluger P.T., Tschop M.H. (2012). Sirtuin 1 and sirtuin 3, physiological modulators of metabolism. Physiol. Rev.

[125] Guarente L. (2013). Calorie restriction and sirtuins revisited. Genes Dev.

[126] Banks A.S., Kon N., Knight C., Matsumoto M., Gutierrez-Juarez R., Rossetti L., Gu W., Accili D. (2008). SirT1 gain of function increases energy efficiency and prevents diabetes in mice. Cell Metab.

[127] Kitada M., Kume S., Kanasaki K., Takeda-Watanabe A., Koya D. (2013). Sirtuins as possible drug targets in type 2 diabetes. Curr. Drug Targets.

[128] Garcia-Rodriguez J.L., Barbier-Torres L., Fernandez-Alvarez S., Juan V.G., Monte M.J., Halilbasic E., Herranz D., Alvarez L., Aspichueta P., Marin J.J. (2014). SIRT1 controls liver regeneration by regulating BA metabolism through FXR and mTOR signaling. Hepatology (Baltim. Md.).

[129] Ruderman N.B., Xu X.J., Nelson L., Cacicedo J.M., Saha A.K., Lan F., Ido Y. (2010). AMPK and SirT1, a long-standing partnership?. Am. J. Physiol. Endocrinol. Metab.

[130] Chen L.L., Deng X.Q., Li N.X. (2007). Effects of calorie restriction on SIRT1 expression in liver of nonalcoholic fatty liver disease: Experiment with rats. Zhonghua Yi Xue Za Zhi.

[131] Deng X.Q., Chen L.L., Li N.X. (2007). The expression of SirT1 in nonalcoholic fatty liver disease induced by high-fat diet in rats. Liver Int.

[132] Colak Y., Ozturk O., Senates E., Tuncer I., Yorulmaz E., Adali G., Doganay L., Enc F.Y. (2011). SirT1 as a potential therapeutic target for treatment of nonalcoholic fatty liver disease. Med. Sci. Monit.

[133] Li Y., Xu S., Giles A., Nakamura K., Lee J.W., Hou X., Donmez G., Li J., Luo Z., Walsh K. (2011). Hepatic overexpression of SirT1 in mice attenuates endoplasmic reticulum stress and insulin resistance in the liver. FASEB J.

[134] Li Y., Wong K., Giles A., Jiang J., Lee J.W., Adams A.C., Kharitonenkov A., Yang Q., Gao B., Guarente L. (2014). Hepatic SirT1 attenuates hepatic steatosis and controls energy balance in mice by inducing fibroblast growth factor 21. Gastroenterology.

[135] Purushotham A., Schug T.T., Xu Q., Surapureddi S., Guo X., Li X. (2009). Hepatocyte-specific deletion of SirT1 alters fatty acid metabolism and results in hepatic steatosis and inflammation. Cell Metab.

[136] Shin S.Y., Kim T.H., Wu H., Choi Y.H., Kim S.G. (2014). SirT1 activation by methylene blue, a repurposed drug, leads to AMPK-mediated inhibition of steatosis and steatohepatitis. Eur. J. Pharmacol.

[137] Lombard D.B., Alt F.W., Cheng H.L., Bunkenborg J., Streeper R.S., Mostoslavsky R., Kim J., Yancopoulos G., Valenzuela D., Murphy A. (2007). Mammalian SirT2 homolog SirT3 regulates global mitochondrial lysine acetylation. Mol. Cell. Biol.

[138] Hebert A.S., Dittenhafer-Reed K.E., Yu W., Bailey D.J., Selen E.S., Boersma M.D., Carson J.J., Tonelli M., Balloon A.J., Higbee A.J. (2013). Calorie restriction and SirT3 trigger global reprogramming of the mitochondrial protein acetylome. Mol. Cell.

[139] Hirschey M.D., Shimazu T., Goetzman E., Jing E., Schwer B., Lombard D.B., Grueter C.A., Harris C., Biddinger S., Ilkayeva O.R. (2010). SirT3 regulates mitochondrial fatty-acid oxidation by reversible enzyme deacetylation. Nature.

[140] Shimazu T., Hirschey M.D., Hua L., Dittenhafer-Reed K.E., Schwer B., Lombard D.B., Li Y., Bunkenborg J., Alt F.W., Denu J.M. (2010). SirT3 deacetylates mitochondrial 3-hydroxy-3- methylglutaryl CoA synthase 2 and regulates ketone body production. Cell Metab.

[141] Fujino T., Kondo J., Ishikawa M., Morikawa K., Yamamoto T.T. (2001). Acetyl-CoA synthetase 2, a mitochondrial matrix enzyme involved in the oxidation of acetate. J. Biol. Chem.

[142] Ahn B.-H., Kim H.-S., Song S., Lee I.H., Liu J., Vassilopoulos A., Deng C.-X., Finkel T. (2008). A role for the mitochondrial deacetylase SirT3 in regulating energy homeostasis. Proc. Natl. Acad. Sci. USA.

[143] Cimen H., Han M.-J., Yang Y., Tong Q., Koc H., Koc E.C. (2009). Regulation of succinate dehydrogenase activity by SirT3 in mammalian mitochondria. Biochemistry.

[144] Finley L.W., Haas W., Desquiret-Dumas V., Wallace D.C., Procaccio V., Gygi S.P., Haigis M.C. (2011). Succinate dehydrogenase is a direct target of sirtuin 3 deacetylase activity. PLoS One.

[145] Zhang D., Liu Z.X., Choi C.S., Tian L., Kibbey R., Dong J., Cline G.W., Wood P.A., Shulman G.I. (2007). Mitochondrial dysfunction due to long-chain acyl-CoA dehydrogenase deficiency causes hepatic steatosis and hepatic insulin resistance. Proc. Natl. Acad. Sci. USA.

[146] Kurtz D.M., Rinaldo P., Rhead W.J., Tian L., Millington D.S., Vockley J., Hamm D.A., Brix A.E., Lindsey J.R., Pinkert C.A. (1998). Targeted disruption of mouse long-chain acyl-CoA dehydrogenase gene reveals crucial roles for fatty acid oxidation. Proc. Natl. Acad. Sci. USA.

[147] Qiu X., Brown K., Hirschey M.D., Verdin E., Chen D. (2010). Calorie restriction reduces oxidative stress by SirT3-mediated SOD2 activation. Cell Metab.

[148] Hirschey M.D., Shimazu T., Huang J.Y., Schwer B., Verdin E. (2011). SirT3 regulates mitochondrial protein acetylation and intermediary metabolism. Cold Spring Harb. Symp. Quant. Biol.

[149] Petro A.E., Cotter J., Cooper D.A., Peters J.C., Surwit S.J., Surwit R.S. (2004). Fat, carbohydrate, and calories in the development of diabetes and obesity in the C57BL/6J mouse. Metab. Clin. Exp.

[150] Rossmeisl M., Rim J.S., Koza R.A., Kozak L.P. (2003). Variation in type 2 diabetes—Related traits in mouse strains susceptible to diet-induced obesity. Diabetes.

[151] Surwit R.S., Feinglos M.N., Rodin J., Sutherland A., Petro A.E., Opara E.C., Kuhn C.M., Rebuffe-Scrive M. (1995). Differential effects of fat and sucrose on the development of obesity and diabetes in C57BL/6J and A/J mice. Metab. Clin. Exp.

[152] Kendrick A.A., Choudhury M., Rahman S.M., McCurdy C.E., Friederich M., van Hove J.L., Watson P.A., Birdsey N., Bao J., Gius D. (2011). Fatty liver is associated with reduced SirT3 activity and mitochondrial protein hyperacetylation. Biochem. J.

[153] Crunkhorn S., Dearie F., Mantzoros C., Gami H., da Silva W.S., Espinoza D., Faucette R., Barry K., Bianco A.C., Patti M.E. (2007). Peroxisome proliferator activator receptor gamma coactivator-1 expression is reduced in obesity: Potential pathogenic role of saturated fatty acids and p38 mitogen-activated protein kinase activation. J. Biol. Chem.

[154] Ji H., Friedman M.I. (2007). Reduced capacity for fatty acid oxidation in rats with inherited susceptibility to diet-induced obesity. Metab. Clin. Exp.

[155] Jing E., Emanuelli B., Hirschey M.D., Boucher J., Lee K.Y., Lombard D., Verdin E.M., Kahn C.R. (2011). Sirtuin-3 (SirT3) regulates skeletal muscle metabolism and insulin signaling via altered mitochondrial oxidation and reactive oxygen species production. Proc. Natl. Acad. Sci. USA.

[156] Bao J., Scott I., Lu Z., Pang L., Dimond C.C., Gius D., Sack M.N. (2010). SirT3 is regulated by nutrient excess and modulates hepatic susceptibility to lipotoxicity. Free Radic. Biol. Med.

[157] Mailloux R.J., Beriault R., Lemire J., Singh R., Chenier D.R., Hamel R.D., Appanna V.D. (2007). The tricarboxylic acid cycle, an ancient metabolic network with a novel twist. PLoS One.

[158] Someya S., Yu W., Hallows W.C., Xu J., Vann J.M., Leeuwenburgh C., Tanokura M., Denu J.M., Prolla T.A. (2010). SirT3 mediates reduction of oxidative damage and prevention of age-related hearing loss under caloric restriction. Cell.

[159] Yu H.S., Oyama T., Isse T., Kitagawa K., Pham T.T., Tanaka M., Kawamoto T. (2010). Formation of acetaldehyde-derived DNA adducts due to alcohol exposure. Chem. Biol. Interact.

[160] Spitz D.R., Oberley L.W. (1989). An assay for superoxide dismutase activity in mammalian tissue homogenates. Anal. Biochem.

[161] Tao R., Coleman M.C., Pennington J.D., Ozden O., Park S.-H., Jiang H., Kim H.-S., Flynn C.R., Hill S., Hayes McDonald W. (2010). SirT3-mediated deacetylation of evolutionarily conserved lysine 122 regulates MnSOD activity in response to stress. Mol. Cell.

[162] He J., Hu B., Shi X., Weidert E.R., Lu P., Xu M., Huang M., Kelley E.E., Xie W. (2013). Activation of the aryl hydrocarbon receptor sensitizes mice to nonalcoholic steatohepatitis by deactivating mitochondrial sirtuin deacetylase SirT3. Mol. Cell. Biol.

[163] Amir M., Czaja M.J. (2011). Autophagy in nonalcoholic steatohepatitis. Expert Rev. Gastroenterol. Hepatol.

[164] Singh R., Kaushik S., Wang Y., Xiang Y., Novak I., Komatsu M., Tanaka K., Cuervo A.M., Czaja M.J. (2009). Autophagy regulates lipid metabolism. Nature.

[165] Yang L., Li P., Fu S., Calay E.S., Hotamisligil G.S. (2010). Defective hepatic autophagy in obesity promotes ER stress and causes insulin resistance. Cell Metab.

[166] Scarpulla R.C., Vega R.B., Kelly D.P. (2012). Transcriptional integration of mitochondrial biogenesis. Trends Endocrinol. Metab.

[167] Aatsinki S.M., Buler M., Salomaki H., Koulu M., Pavek P., Hakkola J. (2014). Metformin induces PGC-1α expression and selectively affects hepatic PGC-1α functions. Br. J. Pharmacol.

[168] Srivastava S., Diaz F., Iommarini L., Aure K., Lombes A., Moraes C.T. (2009). PGC-1α/β induced expression partially compensates for respiratory chain defects in cells from patients with mitochondrial disorders. Hum. Mol. Genet.

[169] Kong X., Wang R., Xue Y., Liu X., Zhang H., Chen Y., Fang F., Chang Y. (2010). Sirtuin 3, a new target of PGC-1α, plays an important role in the suppression of ROS and mitochondrial biogenesis. PLoS One.

[170] St-Pierre J., Drori S., Uldry M., Silvaggi J.M., Rhee J., Jager S., Handschin C., Zheng K., Lin J., Yang W. (2006). Suppression of reactive oxygen species and neurodegeneration by the PGC-1 transcriptional coactivators. Cell.

[171] Giralt A., Hondares E., Villena J.A., Ribas F., Díaz-Delfín J., Giralt M., Iglesias R., Villarroya F. (2011). Peroxisome proliferator-activated receptor-γ coactivator-1α controls transcription of the *SirT3* Gene, an essential component of the thermogenic brown adipocyte phenotype. J. Biol. Chem.

[172] Kubli D.A., Gustafsson Å.B. (2012). Mitochondria and mitophagy: The yin and yang of cell death control. Circ. Res.

[173] Babbar M., Sheikh M.S. (2013). Metabolic stress and disorders related to alterations in mitochondrial fission or fusion. Mol. Cell. Pharmacol.

[174] Palikaras K., Tavernarakis N. (2014). Mitochondrial homeostasis: The interplay between mitophagy and mitochondrial biogenesis. Exp. Gerontol.

[175] Van der Bliek A.M., Shen Q., Kawajiri S. (2013). Mechanisms of mitochondrial fission and fusion. Cold Spring Harb. Perspect. Biol.

[176] Geisler S., Holmstrom K.M., Treis A., Skujat D., Weber S.S., Fiesel F.C., Kahle P.J., Springer W. (2010). The PINK1/Parkin-mediated mitophagy is compromised by PD-associated mutations. Autophagy.

[177] Geisler S., Holmstrom K.M., Skujat D., Fiesel F.C., Rothfuss O.C., Kahle P.J., Springer W. (2010). PINK1/Parkin-mediated mitophagy is dependent on VDAC1 and p62/SQSTM1. Nat. Cell Biol.

[178] Jin S.M., Youle R.J. (2012). PINK1- and Parkin-mediated mitophagy at a glance. J. Cell Sci.

[179] Chan N.C., Salazar A.M., Pham A.H., Sweredoski M.J., Kolawa N.J., Graham R.L.J., Hess S., Chan D.C. (2011). Broad activation of the ubiquitin–proteasome system by Parkin is critical for mitophagy. Hum. Mol. Genet.

[180] Poole A.C., Thomas R.E., Yu S., Vincow E.S., Pallanck L. (2010). The mitochondrial fusion-promoting factor mitofusin is a substrate of the PINK1/Parkin pathway. PLoS One.

[181] Gegg M.E., Cooper J.M., Chau K.-Y., Rojo M., Schapira A.H.V., Taanman J.-W. (2010). Mitofusin 1 and mitofusin 2 are ubiquitinated in a PINK1/Parkin-dependent manner upon induction of mitophagy. Hum. Mol. Genet.

[182] Bhat G., Baba C.S., Pandey A., Kumari N., Choudhuri G. (2012). Life style modification improves insulin resistance and liver histology in patients with non-alcoholic fatty liver disease. World J. Hepatol.

[183] Browning J.D., Baker J.A., Rogers T., Davis J., Satapati S., Burgess S.C. (2011). Short-term weight loss and hepatic triglyceride reduction: Evidence of a metabolic advantage with dietary carbohydrate restriction. Am. J. Clin. Nutr.

[184] Elias M.C., Parise E.R., de Carvalho L., Szejnfeld D., Netto J.P. (2010). Effect of 6-month nutritional intervention on non-alcoholic fatty liver disease. Nutrition.

[185] Goncalves I.O., Oliveira P.J., Ascensao A., Magalhaes J. (2013). Exercise as a therapeutic tool to prevent mitochondrial degeneration in nonalcoholic steatohepatitis. Eur. J. Clin. Investig.

[186] Goncalves I.O., Passos E., Rocha-Rodrigues S., Diogo C.V., Torrella J.R., Rizo D., Viscor G., Santos-Alves E., Marques-Aleixo I., Oliveira P.J. (2014). Physical exercise prevents and mitigates non-alcoholic steatohepatitis-induced liver mitochondrial structural and bioenergetics impairments. Mitochondrion.

[187] Haus J.M., Solomon T.P., Kelly K.R., Fealy C.E., Kullman E.L., Scelsi A.R., Lu L., Pagadala M.R., McCullough A.J., Flask C.A. (2013). Improved hepatic lipid composition following short-term exercise in nonalcoholic fatty liver disease. J. Clin. Endocrinol. Metab.

[188] He Y., Zhang H., Fu F. (2008). The effects of swimming exercise on high-fat-diet-induced steatohepatitis. J. Sports Med. Phys. Fit.

[189] Larson-Meyer D.E., Newcomer B.R., Heilbronn L.K., Volaufova J., Smith S.R., Alfonso A.J., Lefevre M., Rood J.C., Williamson D.A., Ravussin E. (2008). Effect of 6-month calorie restriction and exercise on serum and liver lipids and markers of liver function. Obesity (Silver Spring. Md.).

[190] Rector R.S., Thyfault J.P., Morris R.T., Laye M.J., Borengasser S.J., Booth F.W., Ibdah J.A. (2008). Daily exercise increases hepatic fatty acid oxidation and prevents steatosis in otsuka long-evans tokushima Fatty rats. Am. J. Physiol. Gastrointest. Liver Physiol.

[191] Pacana T., Sanyal A.J. (2012). Vitamin E and nonalcoholic fatty liver disease. Curr. Opin. Clin. Nutr. Metab. Care.

[192] Linden M.A., Fletcher J.A., Morris E.M., Meers G.M., Kearney M.L., Crissey J.M., Laughlin M.H., Booth F.W., Sowers J.R., Ibdah J.A. (2014). Combining metformin and aerobic exercise training in the treatment of type 2 diabetes and NAFLD in OLETF rats. Am. J. Physiol. Endocrinol. Metab.

[193] Chachay V.S., Macdonald G.A., Martin J.H., Whitehead J.P., O’Moore-Sullivan T.M., Lee P., Franklin M., Klein K., Taylor P.J., Ferguson M. (2014). Resveratrol does not benefit patients with non-alcoholic fatty liver disease. Clin. Gastroenterol. Hepatol.

[194] Poulsen M.M., Larsen J.O., Hamilton-Dutoit S., Clasen B.F., Jessen N., Paulsen S.K., Kjaer T.N., Richelsen B., Pedersen S.B. (2012). Resveratrol up-regulates hepatic uncoupling protein 2 and prevents development of nonalcoholic fatty liver disease in rats fed a high-fat diet. Nutr. Res.

[195] Poulsen M.M., Vestergaard P.F., Clasen B.F., Radko Y., Christensen L.P., Stodkilde-Jorgensen H., Moller N., Jessen N., Pedersen S.B., Jorgensen J.O. (2013). High-dose resveratrol supplementation in obese men: An investigator-initiated, randomized, placebo-controlled clinical trial of substrate metabolism, insulin sensitivity, and body composition. Diabetes.

[196] Timmers S., Konings E., Bilet L., Houtkooper R.H., van de Weijer T., Goossens G.H., Hoeks J., van der Krieken S., Ryu D., Kersten S. (2011). Calorie restriction-like effects of 30 days of resveratrol supplementation on energy metabolism and metabolic profile in obese humans. Cell Metab.

[197] Teodoro J.S., Duarte F.V., Gomes A.P., Varela A.T., Peixoto F.M., Rolo A.P., Palmeira C.M. (2013). Berberine reverts hepatic mitochondrial dysfunction in high-fat fed rats: A possible role for SirT3 activation. Mitochondrion.

